# *Escherichia coli* Specific Virulence-Gene Markers Analysis for Quality Control of Ovine Cheese in Slovakia

**DOI:** 10.3390/microorganisms9091808

**Published:** 2021-08-25

**Authors:** Dobroslava Bujňáková, Lívia Karahutová, Vladimír Kmeť

**Affiliations:** Centre of Biosciences of the Slovak Academy of Sciences, Institute of Animal Physiology, 04001 Košice, Slovakia; karahutova@saske.sk (L.K.); kmetv@saske.sk (V.K.)

**Keywords:** ExPEC, STEC, pathogenic potential, phylogenetic grouping, PCR

## Abstract

Shiga toxin-producing and extra-intestinal pathogenic *Escherichia coli* (*E. coli*) have the potential to spread through faecal waste, resulting in contamination of food and causing foodborne disease outbreaks. With the aim of characterizing unpasteurized ovine cheese in Slovakia, a total of 92 *E. coli* strains were examined for eleven representative virulence genes typical for (extra-)intestinal pathogenic *E. coli* and phylogenetic grouping. Phylogenetic groups B1 (36%) and A (32%) were the most dominant, followed by groups C (14%) and D (13%), while the lowest incidence was recorded for F (4%), and E (1%), and 43 (47%) samples carried at least one virulent gene, i.e., potential pathogens. Isolates present in groups E, F and D showed higher presence of virulence genes (100%, 75%, and 67%), versus 55%, 39%, and 28% in commensal B1, C, and A, respectively. Occurrence of *papC* and *fyuA* (both 24%) was highest, followed by *tsh*, *iss*, *stx2*, *cnf1*, *kpsII*, *cvaC*, *stx1*, *iutA* and *eaeA.* Nine *E. coli* strains (almost 10% of all tested and around 21% of our virulence-gene-associated isolates) harboured *stx1*, *stx2* or *eae.* Ovine cheeses in Slovakia are highly contaminated with *E. coli* including potentially pathogenic strains capable of causing intestinal and/or extra-intestinal diseases, and thus may pose a threat to public health while unpasteurized.

## 1. Introduction

Public health hazards associated with consumption of unpasteurized milk products, including related foodborne disease outbreaks, have been reported in parts of the world [1,2], and consequently strict control of the microbial quality of these types of food is required.

Dairy products can be contaminated with various bacteria. Among the pathogenic agents, *Listeria monocytogenes*, *Salmonella* spp., Shiga toxin-producing *E. coli* (STEC) serotype O157:H7 and enterotoxin-producing *Staphylococcus aureus* are most involved in foodborne outbreaks related to the consumption of raw milk cheese in industrialized countries [3]. These foodborne pathogens usually cause illness with acute symptoms restricted to the gastrointestinal tract. However, in some cases, they can cause serious extra-intestinal diseases such as Hemolytic Uremic Syndrome (HUS) associated with *E. coli* O157:H7 [4].

The authors Cancino-Padilla et al. [5] compiled a summary of outbreaks associated with the consumption of various dairy products in the world. Several outbreaks have been associated with the consumption of cheese and other ready to-eat foods concerning their main contaminant *E. coli* stemming from faecal or farm environmental contamination during the milking process [6,7].

The presence of *E. coli* as a reliable indicator of direct or indirect poisoning testifies not only to poor hygienic practices but also (and worse) it can be a source of virulence genes rendering the bacteria pathogenic with the ability to cause a variety of sicknesses. *E. coli* associated with animal or human diseases are divided into two major groups, intestinal and extra-intestinal, and based on their virulence properties into several subgroups (intestinal: enteropathogenic *E. coli* (EPEC), enterohemorrhagic *E. coli* (EHEC) or Shiga toxin-producing *E. coli* (STEC), enterotoxigenic *E. coli* (ETEC), enteroinvasive *E. coli* (EIEC), enteroaggregative *E. coli* (EAEC), and diffusely adherent *E. coli* (DAEC); extra-intestinal: uropathogenic *E. coli* (UPEC), avian pathogenic *E. coli* (APEC), meningitis-associated *E. coli* (MNEC) and necrotoxigenic *E. coli* (NTEC)) [8]. Moreover, virulence-hybrid *E. coli* isolates have also been reported [9,10].

Virulence factors (VF) related to the pathogenicity of various subtypes of *E. coli* are numerous and have a wide range of activity, from those enabled for bacteria colonization to those causing virulence, including adhesins, toxins, iron acquisition factors and polysaccharide capsules, which are usually encoded on pathogenicity islands (PAIs), plasmids and other mobile genetic elements. The most important PAI in Enterobacterales is High Pathogenicity Island, encoded primarily in the iron-uptake system essential for enhancing bacterial condition, the so-called “fitness cost”. Iron uptake as an important survival factor, along with resistance and virulence, allows bacteria to establish in a competitive environment [11].

Since healthy adult cattle and sheep are an asymptomatic reservoir of STEC, faecal contaminating *E. coli* strains isolated from milk and dairy products often contain high prevalence of Vero or Shiga-toxins (*vtx1* and *vtx2* or *stx1* and *stx2*), intimin (*eaeA*) and hemolysin (*hlyA*). These factors can cause bacterial adhesion and invasion into the intestinal epithelial cells, causing severe attaching-effacing (A/E) lesions. Certain STEC occurrence in raw milk, in the particular serotype O157:H7, is well documented and represents a concern since up to 10% of people infected with these bacteria develop HUS, which is potentially a lethal condition, especially in children, immune-deficient and elderly people [12,13]. In 2018, EFSA (European Food Safety Authority) reported a total of 5079 foodborne outbreaks of which 14, involving 775 people, were linked to cheese consumption, and the most common causative agents were STEC and bacterial toxins [14]. The results obtained so far point to the importance of food vehicles in the diffusion of STEC infections at the EU level. Analysis of the virulence gene profiles of the isolated STEC strains highlights the presence of STEC in food with potential for causing severe disease. EFSA recommends reporting on the STEC virulence genes as their analysis represents the basis for molecular risk assessment and the most valuable tool for predicting that risk and the severity of STEC infections in humans.

In recent years, investigators have hypothesized that food, including unpasteurized milk products, can be a reservoir for many other virulence factors responsible for extra-intestinal infections, and can play a major role in the transmission of ExPEC (Extraintestinal pathogenic *Escherichia coli*) strains [2,15,16]. Although research has been ongoing for many years, specific criteria for classifying *E. coli* strains such as ExPEC have not so far been established. According to results obtained by Johnson and Russo [17], ExPEC were defined as *E. coli* isolates containing two or more virulence markers, which were identified by means of multiplex PCR reaction, including *papA* genes (a structural subunit of P-fimbriae) and/or *papC* (P fimbriae), *sfa*/*foc* (S and F1C fimbriae subunits), *afa*/*dra* (adhesins binding antigen Dr), *kpsMT II* (group 2 capsular polysaccharides) and *iutA* (aerobactin receptor) [18]. However, for many bacterial pathogens infecting mucosal tissues, expression of only one specific adhesin is critical and sufficient for pathogenesis. Disruption of the ability of a bacterial pathogen to attach and to colonize a specific tissue by adhesin-mediated receptor recognition is often enough to make it avirulent [2].

Bearing in mind that the possibility of transmission of various pathogenic intestinal and extra-intestinal *E. coli* strains to humans, causing diseases through consumption of raw milk as well as raw milk products, has been reported worldwide [19,20], monitoring not only of *E. coli* presence, but also their pathogenic potential through virulence genes occurrence, becomes a significant necessity. With regard to this development and due to the paucity of data on the microbial contamination of cheese, mainly concerning virulence-associated genes and phylogenetic distribution in Slovakia, we examined 92 *E. coli* isolates from unpasteurized ovine cheeses for possession of traits associated with the virulence of human extra-intestinal pathogenic *E. coli* (ExPEC) or intestinal Vero (Shiga) toxin (*Vtx* or *Stx*)-producing *E. coli* (VTEC or STEC): e.g., *iutA*, *iss*, *cvaC*, *kpsII*, *tsh*, *papC*, *fyuA*, *cnf1*, *stx1*, *stx2*, *eaeA* and phylogenetic grouping.

## 2. Materials and Methods

### 2.1. Source of Samples and Isolation

During the course of one year (August 2018–August 2019), 92 samples of ovine cheese were obtained from local farmers in western Slovakia. The samples were collected in sterile sample collection bags transferred to the laboratory in a cool-box. All cheese samples showed normal physical character including consistency, odour and colour. Ten grams of each cheese sample were homogenized in a Stomacher (BioTech, Prague, Czech Republic) with 90 mL of sterile buffered peptone water (Oxoid, Basingstoke, UK). After preparing the 1:10 dilution, samples were streaked onto Mac Conkey Agar (Oxoid, Basingstoke, UK) and UriSelect Agar (Bio-Rad Laboratories, Hercules, CA, USA) overnight at 37 °C. The typical colonies on both agars were isolated, identified and confirmed as *E. coli* using MALDI-TOF MS biotyper (Bruker Daltonics, Bremen, Germany) [21] and ENTEROtest24 (Erba Lachema Brno, Czech Republic). A single colony of *E. coli* was isolated from each sample.

### 2.2. DNA Preparation

The cultures were inoculated on Nutrient Agar (Oxoid, Basingstoke, UK) and incubated at 37 °C for 24 h. A bacteriological loop was used to carry part of the colony into 100 µL distilled water, which was then vortexed, boiled for 10 min and centrifuged for 2 min at 12,000× *g*, and the supernatant was used for PCR.

### 2.3. Phylogenetic Groups

All isolates were assigned to phylogenetic groups (A, B1, C, B2, D, E and F) based on Clermont phylogenetic typing schemes [22]. The protocols are based on amplification of *chuA*, *yjaA*, *arpA*, *TspE4.C2* DNA fragments and additional testing for specific genes in the E (*arpAgpE*) and C (*trpAgpC*) groups. Classification of the strains was performed based on the presence or absence of genes.

### 2.4. Genes of Virulence Factors

All isolates were subjected to a multiplex and/or single PCR analysis for detection genes associated with ExPEC, specifically: *iutA*–receptor for aerobactin, *cvaC*–colicin V, *kpsII*–capsular polysialic acid virulence factor, *iss*–increased serum survival, *tsh*–temperature sensitive haemagglutinin, *papC*–P fimbrial adhesin and *fyuA*–yersiniabactin receptor for ferric yersiniabactin uptake. Moreover, we used genes encoding Shiga-toxin types 1, 2 (*stx1*, *stx2*), cytotoxic necrotizing factor (CNF1) and the gene encoding intimin for attaching and effacing mechanisms (*eaeA*). PCR primers, length of their amplified products, annealing and references are listed in Table 1. The amplifications were carried out in a single tube with a volume of 25 μL, utilizing TaqI polymerase (Solis Biodyne, Tartu, Estonia). Amplified products were run on 1.5% agarose gel.

## 3. Results

### 3.1. Phylogenetic Grouping

Phylogenetic grouping of 92 *E. coli* isolates from unpasteurized cheese showed that 33 (35.87%), 29 (31.52%), and 13 (14.13%) belonged in phylogenetic groups B1, A, and C respectively, as shown in Table 2, and these were followed by group D (12 isolates; 13.04%). Finally, groups E and F were less prevalent with 1.09% (1 isolate) and 4.35% (4 isolates), while group B2 was not detected.

Phylogenetic groups B1 and A were predominant among the tested isolates. It is interesting to note that 54.6% (18/33) of isolates belonging in group B1 as well as 100% (1/1), 75% (3/4) and 66.6% (8/12) of isolates belonging in groups E, F and D respectively, were found to have at least one of the examined virulence genes (four isolates contained three virulence genes—*iss*, *cvaC*, *papC*—group B1; *iss*, *iutA*, *cnf1*- group D; *stx1*, *stx2*, *cnf1–*group B1 and *iss*, *cvaC*, *cnf1*-group C) (Table 3 and Table 4).

### 3.2. The Occurrence of E. coli and Virulence Genes in Unpasteurized Cheese

All 92 samples of ovine cheese collected from various locations in western Slovakia were contaminated with *E. coli*. Isolates were investigated for the presence of 11 virulence genes usually present in pathogenic *E. coli*. The occurrence of potentially pathogenic *E. coli* (which carried one or more of the targeted virulence genes) was 47% (*n* = 43). The remaining 49 (53%) isolates were negative. Isolates present in groups E, F and D showed the highest presence of virulence genes (100%, 75% and 67% respectively); however phylogenetic groups considered as commensal, i.e., B1, C and A, also had a high incidence of virulence genes (55%, 39% and 28% respectively). It was found that the most prevalent genes among 43 isolates with virulence factors were *papC* and *fyuA* detected in 11 isolates (both 51%), while 10 (23%), 7 (16%), 6 (14%), 6 (14%), 5 (12%), 4 (9%), 3 (7%), 2 (4.6%) and 1 (2.3%) isolates were positive for *tsh*, *iss*, *stx2*, *cnf1*, *kpsII*, *cvaC*, *stx1*, *iutA* and *eaeA* genes, respectively (Table 3).

Based on the distribution of the various virulence genes investigated, all the tested isolates exhibited 28 different virulence gene patterns. Four isolates were positive for three targeted virulence genes, 14 showed presence of two genes, and in 26 others one gene was detected.

The three most prevalent patterns consisted of isolates possessing one virulence gene, *fyuA*, *tsh*, or *papC* (*n* = 6, *n* = 6, *n* = 6 and *n* = 5 respectively). Based on the distribution of verotoxines and intimin, nine of the tested isolates exhibited a positive signal for one of those genes. Specifically, five isolates harbored clinically and epidemiologically the most important *stx2* alone, two only *stx1*, and one strain featured both *stx1* and *stx2* genes together with gene cnf1. The gene encoding cytotoxic necrotizing factor (CNF1) was detected in six isolates. Only one of the isolates was positive for *eae*, which could be classified as EPEC defined as *Stx*-negative *E. coli* able to produce A/E lesions on intestinal cells. Detailed results are shown in Table 4.

## 4. Discussion

Foodborne diseases caused by various bacterial pathogens are a significant global public health concern. Based on the above, the EU has recently strengthened the rules for strict control of food safety and public health to cope with the spread of certain infectious diseases.

The presence of *E. coli* in milk and milk products is an important indicator of faecal or environmental contamination and poor hygienic practices. Conventional microbiological diagnostics in food control include only determination of *E. coli* numbers per gram without any other characterization of isolated strains.

In addition to the presence of *E. coli* denoting faecal contamination, the presence of virulence-related genes in *E. coli* strains refers to the pathogenicity of the isolates. Previous studies documented the equation of some *E. coli* isolates from raw milk and products for virulence markers [2,19,29]. Considering the above, the present study thus aimed to assess raw cheese quality by capturing live *E. coli* and investigating the *E. coli* isolates for certain functioning virulence-associated genes using PCR assay.

Based on our results it is clear that single or double virulence factors were demonstrated in the majority of examined isolates. One isolate carried two virulence factors *papC* and *kpsII*, which were described by Johnson and Russo [17] as ExPEC, but the importance of this remains irrelevant regarding the possibility of the presence of other markers, which were not the subject of this study.

In our project, every sample of the 92 cheeses was contaminated with *E. coli.* A similar high prevalence of *E. coli* in cheese has also been reported in Brazil (96–97.7%) [20]. Altogether, 47% (43/92) of the investigated dairy products carried potentially pathogenic *E. coli* (possessing one or more of the virulence genes tested), as shown in Table 3.

When interpreting the results, keep in mind that the data of the various diagnostic procedures are not directly comparable due to differences in strategy sampling and analytical application method. Our approach used for selecting the colonies forming the dataset was based on the selection of only a single colony from every sample. A different approach was used by Skočková et al. [30] for monitoring of the occurrence of STEC in swabs from the carcasses of pigs and cattle at slaughterhouses in the Czech Republic. After homogenization and incubation, 1 mL of bacterial suspension was used for DNA isolation, and after PCR, *stx*-positive samples were inoculated on selective media, incubated overnight and up to 50 colonies from one sample with *E. coli* morphology were investigated for the presence of *stx* and *eaeA* genes. Their results showed the prevalence of STEC in cattle (3.9%) and pig (5.1%) carcasses in the Czech Republic, although raw meat has not been considered an important source of STEC.

From our results concerning *stx* and *eae*, nine *E. coli* strains (almost 10% of all tested and around 21% of our virulence gene-associated isolates) harboured *stx1*, *stx2* or *eae* (Table 4). Of these, five contained *stx2* alone, and as described by Friedrich et al. [31], *stx2* is clinically and epidemiologically the most important Shiga-toxin type, and the probability of HUS development in infections from strains harbouring *stx2* is higher than that from strains containing either *stx1* or both *stx1* and *stx2*. Healthy sheep are asymptomatic reservoirs of STEC and thus faecal contamination during milk processing explains the occurrence of these bacteria in dairy products, and this is a significant problem not only for fresh cheese, but also for hard ripened cheeses, because of the ability of STEC to survive during the production procedure with periods of maturation.

In the period 2012–2017, a total of 330 STEC outbreaks were reported in 18 countries of the European Union, involving 2841 cases, 463 hospitalizations and five fatalities. Of all these outbreaks reported, the food vehicle was identified for 164 outbreaks (49.7%) and five outbreaks by strength of evidence of identification of food vehicle were reported from Slovakia and Poland [32]. In 2018, STEC diseases were the fourth most common zoonosis in Europe.

The report on zoonosis in Slovakia indicates, that in 2016, from 46 examined food samples (cheeses from unpasteurized milk, meat products), STECs were confirmed in 28.26%. A year later, twenty-six foods were examined with only one being STEC-positive. Reports from the following years (2018–2020) did not contain data on the STEC situation in Slovakia (https://www.mpsr.sk/?navID=47&sID=111&navID2=506, accessed on 12 December 2016).

Other important virulence determinants include the locus of enterocyte effacement (LEE) shared by EPEC. This 35–45 kb pathogenicity island is responsible for the formation of attaching and effacing (A/E) lesions on intestinal epithelial cells. It contains the *eaeA* gene encoding the outer membrane adhesin, intimin, which mediates tight contact between STEC or EPEC and intestinal epithelial cells. The *eaeA* gene is a well-known virulence factor not only for EPEC and EHEC but also atypical EPEC, in which the *eaeA* gene occurs alone without the presence of adherence factor plasmid (*pEAF*) genes [8]. Our study identified the *eaeA* gene in one isolate (2.3%) (Table 3 and Table 4), which could be classified as EPEC, defined as a *Stx*-negative *E. coli* strain able to produce A/E lesions on intestinal cells, detectable in vitro by means of positive *eae* PCR testing. Our result showing the occurrence of the *eae* gene is in agreement with several previous studies: 9.1% in Saudi Arabia [19], 3.1% in Slovakia [29] and 0.9% in Egypt [2].

Since none of our strains carried *stx* and *eae* together, the strains would not have to be able to adhere to the cells and produce Shiga toxins. However, the EFSA Journal [32] notes, inter alia, that the presence of intimin (*eae* gene) was an aggravating factor, but this virulence factor was not always essential for severe illness, suggesting that there is an alternative mechanism of attachment. As an example, during 2011, a large outbreak caused by an unusual *E. coli* strain was reported in Germany. The pathotype combined the virulence potential of STEC and enteroaggregative *E. coli*. The aggregative adherence fimbriae colonization mechanism substituted for the locus of enterocyte effacement functions normally encoded by the *eae* gene in EHEC strains. Clinical presentation of the infection also included bloody diarrhea and HUS [33]. The Cytotoxic Necrotizing Factor (CNF1) [28] is a bacterial virulence factor associated with ExPEC strains causing urinary tract infection and meningitis that induces a drastic rebuilding of the microfilamental network on various eukaryotic cells in culture into thick stress fibers. The *cnf1* was detected in six (6.5%) of all tested and around 14% of our virulence-gene-associated isolates. The incidence of the *cnf1* virulence marker in *E. coli* strains isolated from traditional Slovak ovine cheese was also described by Holko et al. [29], and *cnf1* was confirmed in 3 isolates from 95 (3%) examined overall.

Other occurring virulence-associated traits were genes for adhesins, protectins/serum resistance, iron uptake and toxins: *papC*, *iss*, *cvaC*, *kpsII*, *fyuA*, *iutA* and *tsh-*specific virulence markers associated with extra-intestinal infection. One of the explanations for their occurrence in milk and dairy products could be the possibility of udder infection (pre/sub-clinical mastitis) [34].

Of the adhesion- and iron uptake-encoding virulence factors included in our study, the *papC* and *fyuA* genes were the most frequent. We found 11 *E. coli* harbouring *papC* (P fimbria), a gene which has been associated with upper urinary tract infection [35], and in some isolates was associated with some of the genes under investigation in this study: *iss*, *cvaC*, *kpsII*, *stx1* and/or *stx2*. A combination of P fimbria and Shiga-toxins could be interesting due to the possibility of their causing problems related to urinary tract infection and HUS. Eleven of our isolates expressed *fyuA* (ferric yersiniabactin uptake), a type of siderophore, which was originally detected in *Yersinia pestis*, and contributes to the pathogenicity of UPEC, especially during colonization of the urinary tract. Yersiniabactin may protect bacterial cells against the host immune response [36]. The most important ColV and ColBM virulence plasmids associated with ExPEC virulence includes the aerobactin (*iutA/iucABCD*). This operon encodes high-affinity iron-transport systems, which are used by bacteria to obtain iron in low-iron conditions such as those they encounter in host fluids and tissues. As many as 30% of our virulence-gene-associated isolates carried at least one siderophore gene, which allows these strains to survive in low iron environments, for example in the bladder or other host fluids and tissues.

Another gene found in the core genome of ExPEC large virulence plasmids is *iss*, which encodes a protein linked with increased serum survival in human *E. coli* isolates. Numerous studies have documented its strong alignment with virulent (but not with avirulent) *E. coli* strains [37]. Almost 16% of our virulence-gene-associated isolates exhibited a protection factor against phagocytosis, and moreover, connected either with adhesins or iron uptake genes and/or factors facilitating colonization.

No less important a role during infection is played by toxins, as they contribute to the spreading of bacteria in tissues, increased cytotoxicity and insensitivity to neutrophils. One of the most frequently detected genes encoding a toxin in ExPEC is *tsh* (temperature-sensitive hemagglutinin), a member of the autotransporter group of proteins first identified in avian-pathogenic *Escherichia coli* (APEC). Autotransporters are a family of autonomously secreted proteins from gram-negative bacteria exhibiting diverse functions involved in virulence, including adhesins, proteases, cytotoxins and cell invasion proteins. The above mentioned toxin contributes to the development of lesions and deposition of fibrin in avian air sacs. It can act both as an adhesin and as a serine protease, agglutinating erythrocytes while in contact with the extracellular surface of the bacterial cells. It can adhere to purified haemoglobin and bind with great efficiency to extracellular matrix proteins. It cleaves casein and exhibits mucinolytic activity [38]. This toxin was detected in 23% of our virulence-gene-associated isolates.

The important protectins/serum resistance in ExPEC involve capsula antigens *kpsII* (protection factor against phagocytosis and the spreading factor) and *cvaC* (factor facilitating colonization). In our study, we found their presence together in up to 21% of our virulence-gene-associated isolates.

*E. coli* strains can be classified into the following phylogenetic groups: A, B1, B2, C, D, E, F, and clade I [22]. A link between the virulence of a strain and its phylogenetic group has been previously reported by Clermont et al. [22,39]. Commensal *E. coli*, with no pathogenic features, occurring among other places on the gastrointestinal tract mucosa, most often represent group A or B1. Pathogenic *E. coli* responsible for intestinal infections represent phylogenetic groups A, B1 or D. *E. coli* responsible for extra-intestinal infections belong in groups B2 and D. Group E is related to group D (including O157: H7), while group F is related to the main group B2. Clones of *E. coli* strains, which are genetically diverse but phenotypically indistinguishable, have been assigned to cryptic clade I [40,41].

The phylogenetic grouping examined by us showed that most *E. coli* isolates belonged in groups B1 and A, followed by strains belonging in group D, while groups E and F occurred infrequently as shown in Table 2. Our results are in agreement with those reported by Rúgeles et al. and Ombarak et al. [2,42], in which *E. coli* isolated from foods mainly belonged in A and B1 phylogenetic groups. The potentially virulent strains (43 strains) were mostly classified into phylogenetic group E (100%), followed by group F (75%), group D (67%), group B1 (55%), group C (38.5%) and group A (27.6%) (Table 3).

## 5. Conclusions

Our obtained data suggest that *E. coli* contaminating our cheese products might have the potential for causing intestinal and, moreover, extra-intestinal infections. Although our study consists of a relatively small number of samples and therefore faces limitations in statistical analysis, it provides important information about the phylogenetic background and incidence of virulent *E. coli* isolated from cheese produced from unpasteurized ovine milk in the Slovak Republic. Our results clearly suggest that microbial quality and safety of unpasteurized cheese made from raw ovine milk produced by local farmers and distributors are not sufficiently good. The presence of coliform bacteria in raw milk products indicates not only poor hygiene, but even worse, these microorganisms are vehicles for virulent genes with the potential to cause a variety of intestinal and extra-intestinal diseases in consumers. As a relatively large number of people still consume raw milk and raw milk products, we would emphasize the increased need for food control and ensuring improved hygiene, thereby contributing to the reduction of potential public health threats.

## Figures and Tables

**Table 1:** PCR primers, length of the amplified products, annealing and references.

Gene	Primer Sequence (5′3′)	Product	T_ann_	Reference
*arpA*	AACGCTATTCGCCAGCTTGC	400 bp	59 °C	[22]
	TCTCCCCATACCGTACGCTA			
*chuA*	ATGGTACCGGACGAACCAAC	288 bp	59 °C	[22]
	TGCCGCCAGTACCAAAGACA			
*yjaA*	CAAACGTGAAGTGTCAGGAG	211 bp	59 °C	[22]
	AATGCGTTCCTCAACCTGTG			
*tspE4.C4*	CACTATTCGTAAGGTCATCC	152 bp	59 °C	[22]
	AGTTTATCGCTGCGGGTCGC			
*ArpAgpE.f*	GATTCCATCTTGTCAAAATATGCC	301 bp	57 °C	[23]
	GAAAAGAAAAAGAATTCCCAAGAG			
*trpAgpC.1*	AGTTTTATGCCCAGTGCGAG	219 bp	59 °C	[23]
	TCTGCGCCGGTCACGCCC			
*kps II*	GCGCATTTGCTGATACTGTTG	272 bp	63 °C	[24]
	CATCCAGACGATAAGCATGAGCA			
*iss*	ATCACATAGGATTCTGCCG	700 bp	61 °C	[25]
	ACAAAAAGTTCTATCGCTTCC			
*papC*	GACGGCTGTACTGCAGGGTGTGGCG	328 bp	61 °C	[26]
	ATATCCTTTCTGCAGGGATGCAATA			
*cvaC*	CACACACAAACGGGAGCTGTT	680 bp	63 °C	[24]
	CACACACAAACGGGAGCTGTT			
*tsh*	GGTGGTGCACTGGAGTGG	620 bp	55 °C	[27]
	AGTCCAGCGTGATAGTGG			
*iutA*	GGCTGGACATGGGAACTGG	300 bp	63 °C	[24]
	CGTCGGGAACGGGTAGAATCG			
*fyuA*	TGATTAACCCCGCGACGGGAA	880 bp	55 °C	[24]
	CGCAGTAGGCACGATGTTGTA			
*stx1*	ACGTTACAGCGTGTTGCRGGGATC	121 bp	63 °C	[28]
	TTGCCACAGACTGCGTCAGTRAGG			
*stx2*	TGTGGCTGGGTTCGTTAATACGGC	102 bp	63 °C	[28]
	TCCGTTGTCATGGAAACCGTTGTC			
*eaeA*	TGAGCGGCTGGCATGAGTCATAC	241 bp	63 °C	[28]
	TCGATCCCCATCGTCACCAGAGG			
*cnf1*	GGCGACAAATGCAGTATTGCTTGG	552 bp	63 °C	[28]
	GACGTTGGTTGCGGTAATTTTGGG			

**Table 2:** Phylogenetic distribution of *E. coli* from unpasteurized cheese.

**Phylogenetic Groups**	**No. of Isolates/% of Occurrence** **(*n* = 92)**	**Distribution According to Gene Groupings (*n*)**	**Quadruplex Genotype and Next Step for C or E Phylogroup**					
			*arpA*	*chuA*	*yjaA*	*TspE4.C4*	*ArpA* for E Group	*trpA* for C Group
Group A/C	29/(32%)	12	+					
		17	+		+			-
Group C	13/(14%)	13	+		+			+
Group B1	33/(36%)	33	+			+		
Group D/E	12/(13%)	8	+	+		+	-	
		4	+	+			-	
Group E	1/(1%)	1	+	+	+		+	
Group F	4/(4%)	4		+				

**Table 3:** The percentage of occurrence of virulence-related genes in various phylogenetic groups of *E. coli*.

**Phylogenetic Groups**	**No. of Isolates/No. with Virulence Genes** **(% Virulent Strains)**		**Presence of Virulence Genes**									
		*iss*	*cvaC*	*papC*	*iutA*	*tsh*	*fyuA*	*kpsII*	*stx1*	*stx2*	*eaeA*	*cnf1*
Group A	29/8 (27.6%)			1		1	4	2		2		
Group B1	33/18 (54.6%)	4	3	5	1	6	3		2	3		2
Group C	13/5 (38.5%)	1	1	2		1	1			1		2
Group D	12/8 (66.6%)	1		2	1	2	1	2	1			2
Group E	1/1 (100%)										1	
Group F	4/3 (75%)	1		1			2	1				
TOTAL	92/43 (46.7%)	7	4	11	2	10	11	5	3	6	1	6

**Table 4:** Presence of virulence gene patterns in *E. coli*.

No. of Genes	Virulence Genes	No. of Isolates (*n* = 92)
1	*iss*	1
1	*cvaC*	1
1	*papC*	5
1	*iutA*	1
1	*tsh*	6
1	*fyuA*	6
2	*fyuA*, *cnf*1	1
1	*cnf*1	1
1	*kpsII*	2
3	*iss*, *cvaC*, *cnf*1	1
2	*iss*, *papC*	1
2	*iss*, *cnf*1	1
3	*iss*, *iutA*, *cnf1*	1
2	*iss*, *fyuA*	1
3	*iss*, *cvaC*, *papC*	1
2	*tsh*, *fyuA*	1
2	*tsh*, *kpsII*	1
2	*tsh*, *stx2*	2
2	*papC*, *cvaC*	1
2	*papC*, *kpsII*	1
2	*papC*, *stx1*	1
2	*papC*, *stx2*	1
2	*fyuA*, *kpsII*	1
2	*fyuA*, *stx2*	1
1	*eaeA*	1
1	*stx1*	1
1	*stx2*	1
3	*stx1*, *stx2*, *cnf*1	1
0	No gene	49
